# Microstructures and Properties of Cu-rGO Composites Prepared by Microwave Sintering

**DOI:** 10.3390/ma14174899

**Published:** 2021-08-28

**Authors:** Xuebin Chen, Lei Zhao, Liwu Jiang, Haizhou Wang

**Affiliations:** 1 Beijing Advanced Innovation Center for Materials Genome Engineering, National Center for Materials Service Safety, University of Science and Technology Beijing, Beijing 100083, China; xbinchen@yeah.net; 2 Beijing Key Laboratory of Metal Materials Characterization, The NCS Testing Technology Co., Ltd., Beijing 100081, China; zhaolei@ncschina.com

**Keywords:** materials preparation, microwave sintering, Copper-graphene, microstructure, composite properties

## Abstract

This study investigated the effects of microwave sintering on the microstructures and properties of copper-rGO composites. Graphene oxide was coated onto copper particles by wet ball milling, and copper-rGO composites were formed upon microwave sintering in an argon atmosphere. Scanning electron microscopy was then used to observe the mixing in the ball-milled composite powder, and the morphology of the bulk composite after microwave sintering. Raman spectra revealed how graphene oxide changed with ball milling and with microwave sintering. The microhardness, electrical conductivity, and thermal conductivity of the composite were also measured. The results showed that graphene oxide and copper particles were well combined and uniformly distributed after wet ball milling. The overall microhardness of microwave-sintered samples was 81.1 HV, which was 14.2% greater than that of pure copper (71 HV). After microwave sintering, the microhardness of the samples in areas showing copper oxide precipitates with eutectic structures was 89.5 HV, whereas the microhardness of the precipitate-free areas was 70.6 HV. The electrical conductivity of the samples was 87.10 IACS%, and their thermal conductivity was 391.62 W·m^−1^·K^−1^.

## 1. Introduction

Graphene (Gr) and its family are extensively used as fillers with metals due to their overall extraordinary properties such as excellent strength and hardness, the highest value of thermal conductivity, and larger surface area [1,2]. Compared to graphene, graphene oxide (GO) is more reliable to use as filler due to ease of production on a larger scale and mixing with metals. Different researchers used a variety of techniques to enhance the thermomechanical properties of metal graphene composites such as powder metallurgy [3,4]. In particular, Chu et al. fabricated Cu-rGO composites by using conventional powder metallurgy. They enhanced the yield strength (146 MPa) and ultimate tensile (214 MPa) of composites considerably [5]. Fang et al. fabricated both Cu-rGO and Cu-graphene composites; the mechanical strength and ductility both increased significantly, even above pure Cu [6]. Zhang et al. reported the effect of GNPs and rGO on the thermo-mechanical properties of the Cu matrix. They observed firstly the strength is increased with low concentration [7]. All these characteristics make graphene an ideal material for enhancing the electrical and thermal conductivities of metals, through the formation of high-performance graphene composites. For many functional applications, graphene may be an ideal metal-matrix composite reinforcement, because graphene can improve the mechanical properties such as the hardness of the composites, and also enhance their electrical conductivity, resulting in high-strength and high-conductivity metal matrix composites [8,9,10,11]. Given its unique electrical, mechanical, and thermal properties, graphene is a very promising reinforcement for copper (Cu) matrix composites [2,12,13,14,15,16,17,18,19,20,21].

Owing to their excellent electrical conductivity, Cu alloys are highly popular materials that receive great attention from research institutions in China and overseas. Raj Kumar et al. prepared Cu-graphene composites by microwave sintering, and the microwave-sintered samples were superior to those obtained by traditional processes [22]. To explore the effects of different sintering methods on Cu-graphene composites, Ayyappadas et al. [23] applied different methods to sinter composites and found that the composites were well coupled to the microwave field. The processing cycle was 63% shorter than that of the traditional process. Microstructure analysis revealed a uniform distribution of graphene in the Cu matrix. Kim et al. [24] used ball milling and high-ratio differential speed rolling to synthesize Cu matrix composites reinforced by multilayer graphene with different volume fractions (0.5% and 1.0%). They found that graphene was successfully exfoliated into a nanoscale-dispersed reinforcement phase, and the mechanical properties of the Cu matrix composites were remarkably improved. Zhang et al. [25] found that continuous three-layered graphene could effectively improve both the oxidation resistance of Cu foil in air and its electrochemical corrosion resistance in 0.1 M sodium chloride solution. Paloma et al. [26] comprehensively reviewed the properties of a range of Cu and graphene materials in various conditions, and described the effects of graphene type and content, in addition to the effects of processing conditions. Finally, they discussed the application prospects of Cu-graphene composites. Most studies have shown that the best performance can be achieved when 0.5% GO is used in composites [27,28].

In the present study, graphene oxide (GO) was mixed with Cu particles by mechanical wet ball milling. Bulk graphene-copper composites were prepared by in situ reduction of the GO during microwave sintering of the Cu-GO composite powder in an argon atmosphere. We analyzed the changes in the powder before and after ball milling, and evaluated the effects of microwave sintering on the microstructures and properties of graphene-reinforced Cu matrix composites.

## 2. Materials and Methods

The GO used in the experiment was >97% purity, and approximately 5–10 μm in diameter (Beijing Carbon Century Technology Co., Ltd., Beijing, China). The GO powder had a black color and showed strong hydrophilicity, with some agglomeration. The Cu used in the experiment was electrolytic Cu powder with a particle size of 50 μm and 99.9% purity. Briefly, 0.5 g of GO was weighed into a beaker and 100 mL of ethanol was added. The mixture was stirred to obtain a graphene oxide dispersion. Then, 99.5 g of Cu powder was added to this dispersion, which was milled using stainless steel balls for 8 h. The speed of the ball mill was set to 280 r/min, and the ball-to-material ratio was 8:1. The ball-milled dispersion was dried at 80 °C for 20 h under vacuum. After sieving, a 0.5% Cu-GO composite powder was obtained.

**Figure 1:**
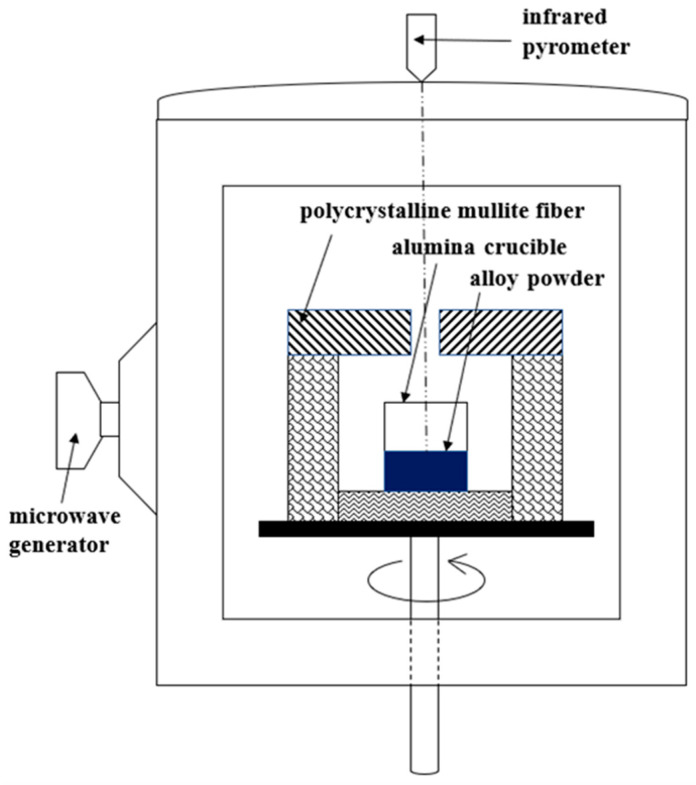
Schematic illustration of microwave-sintering apparatus.

The microwave device used in this study was purchased from Kunming University of Science and Technology, and is shown in Figure 1, where The Key Laboratory of Unconventional Metallurgy, Ministry of Education is the maker of the microwave. The microwave device used in this study is shown in Figure 1. The microwave had a power of 5.5 kW and a frequency of 2.45 GHz. An infrared pyrometer (Raytek MM2MH, Santa Cruz, CA, USA) was used to measure temperature in the range of 300–1300 °C, and the transmitting power of the probe was 0.75. The composite powder was placed in an alumina crucible which was then placed into the microwave cavity, and insulated by polycrystalline mullite and asbestos. Before microwave sintering, the cavity was evacuated to 0.4 MPa and then backfilled with argon. This step was performed three times to prevent oxidation. During the sintering process, the input power of the microwave was manually adjusted to control the temperature ramp rate at 30 °C/min, and sintering was carried out under 99.9% argon flow. The sintering temperature was 900 °C and was held for 40 min. The composite sample was allowed to cool to room temperature before being removed from the microwave device. Microstructural analysis and property tests were carried out after grinding and polishing.

The microstructures of composite samples were first characterized using an optical microscope (LEICA dm-6000m, Leica Instruments Ltd., Shanghai, China). Further characterization of microstructure and composition was carried out using a tungsten-filament scanning electron microscope (SEM; JSM IT-300, JEOL, Akishima, Tokyo, Japan; 20 kV, 1.5 nA, back-scattered electron detector) coupled with energy dispersive spectroscopy (EDS). Raman spectra were measured for the GO powder, ball-milled composite powder, and microwave-prepared composites using a high-resolution Raman spectrometer (LabRAM HR Evolution, HORIBA Jobin Yvon S.A.S, Palaiseau, France; resolution = 0.65 cm^−1^, excitation wavelength = 532 nm, shift range = 100–4000 cm^−1^). Additionally, the microhardness of the samples was measured using a Qness q-10 Vickers microhardness tester (load = 0.5 kg, indentation time = 15 s), and the arithmetic mean of measurements at six points on the sample surface was obtained. The electrical conductivity of the composites was measured using an electrical conductivity tester (SIGMATEST 2.069, FOERSTER, Foerster, Reutlingen, German), and their thermal properties (λ: thermal conductivity; α: thermal diffusion coefficient; C_p_: specific heat) were measured using a TGA-DSC synchronous thermal analyzer (TA Instruments, New Castle, DE, USA; λ: thermal conductivity; α: thermal diffusion coefficient; C_p_: specific heat).

## 3. Results and Discussion

### 3.1. Powder Characterization

**Figure 2:**
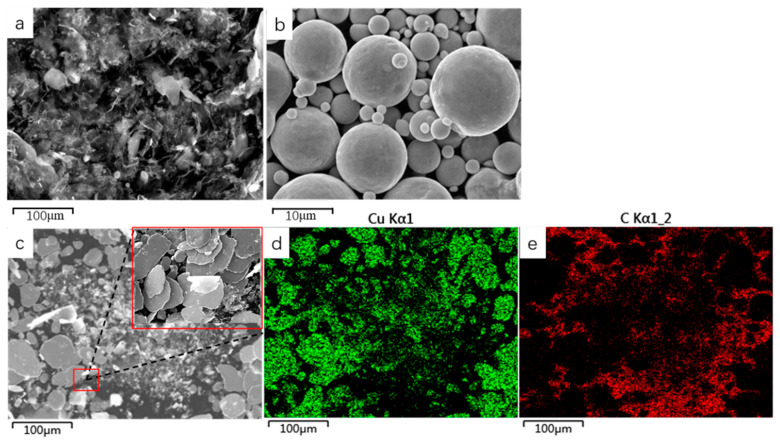
SEM morphological images (**a**: graphene oxide; **b**: Cu powder; **c**: composite powder) and EDS elemental mapping (**d**: Cu; **e**: C).

We used SEM to investigate the distribution of Cu and GO within the composites. Graphene oxide before ball milling (Figure 2a) and Copper powder before ball milling (Figure 2b) The results showed that the Cu particles exhibited plastic deformation after ball milling. These particles gradually formed sheets and their particle size increased during the process of continuous crushing and welding (Figure 2c). The high-magnification image (Figure 2c) shows that the GO had exfoliated into thin sheets, which coated the surface of Cu particles (Figure 2c). To further characterize the mixing of the powder after ball milling, we performed EDS elemental mapping and observed that Cu was only present in the powder particles in the SEM image (Figure 2d). All the Cu particles basically formed sheets during ball milling, and the particle size increased due to welding. In contrast, carbon (C) was generally distributed around the Cu particles (Figure 2e). These results indicated that the Cu powder and GO were fully mixed after ball milling; GO was combined with the Cu matrix and coated on the surface of the particles.

### 3.2. Raman Spectroscopy

**Figure 3:**
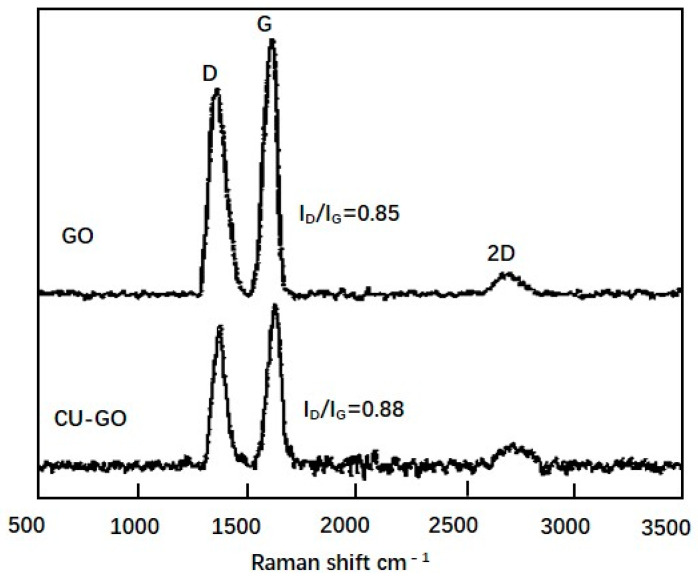
Comparison of Raman spectra of graphene oxide (GO) powder and ball-milled Cu-GO composite powder.

To verify the presence and structural integrity of graphene oxide in the composite powder, we performed Raman spectroscopy. The results showed that the Raman spectra of the starting GO had a distinct D peak and a G peak at 1348 and 1585 cm^−1^, respectively, with an evident 2D peak at 2697 cm^−1^ (Figure 3). GO was usually a highly functionalized graphene derivative. Therefore, the value I_D_/I_G_ was relatively high due to the high intensity of D band, which was ascribed to the generated sp3 carbons owing to the oxidation. Moreover, the high intensities of both peaks indicated the presence of chemical functional groups. Furthermore, due to the multiple layers of GO, the material had relatively more internal defects [27,29].

The ball-milled composite powder had obvious D and G peaks at 1353 and 1598 cm^−1^, respectively, despite their substantially reduced intensity compared with the peaks in the original GO (Figure 3). The obtained I_D_/I_G_ was 0.88, which was close to that of the original GO. This result indicated the GO was thinned by ball milling, but maintained its chemical structure.

**Figure 4:**
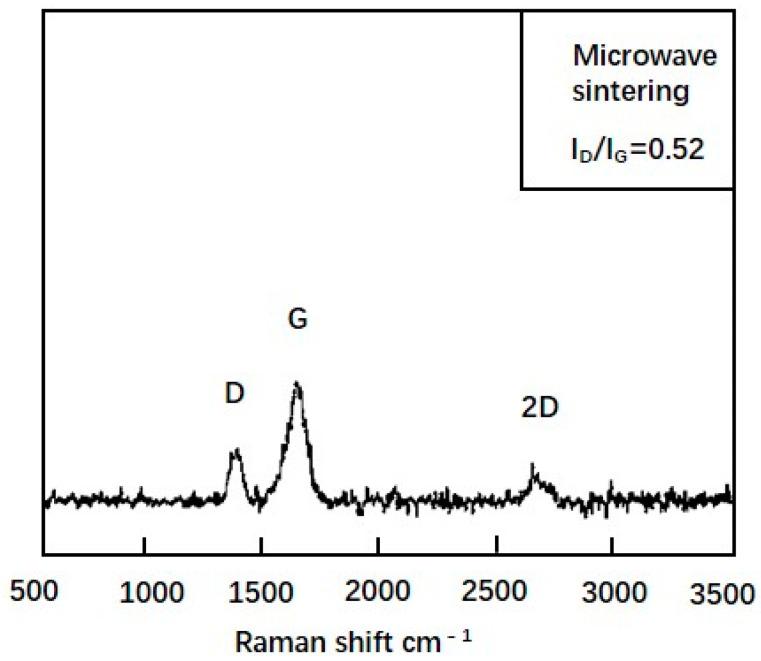
Raman spectra of microwave-prepared bulk composite.

After the composite powder was microwave sintered, we characterized the bulk composites by Raman spectral analysis. After microwave sintering of the composite powder, the D peak and G peak appeared at 1358 and 1605 cm^−1^, respectively, with considerably decreased peak intensity (Figure 4). The I_D_/I_G_ ratio was estimated to be 0.52, which was considerably lower than that for the powder prior to sintering. Compared with the composite powder, internal defects were reduced in the microwave-sintered “graphene oxide”. These observations indicated that GO was in fact reduced to rGO, which contained far fewer chemical functional groups, and therefore had fewer internal defects than GO [27]. Moreover, the increase in 2D band was due to the reduction of GO to rGO.

### 3.3. Composite Morphology

**Figure 5:**
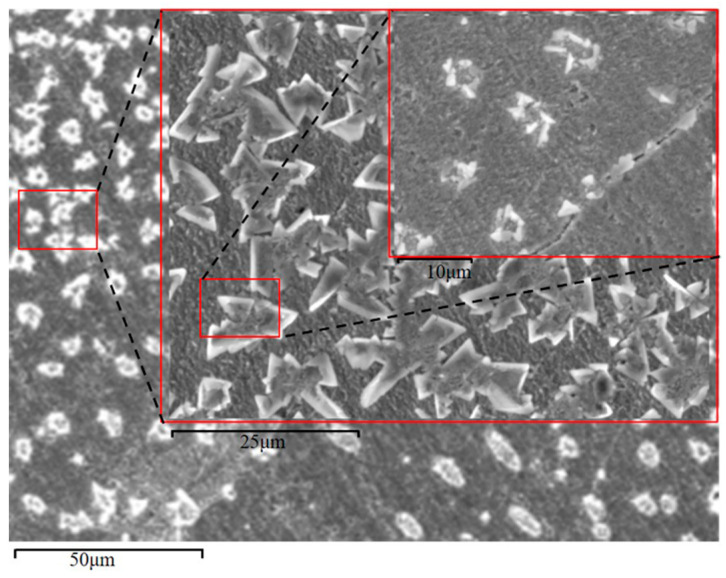
SEM morphology of microwave-sintered Cu-rGO composites.

**Figure 6:**
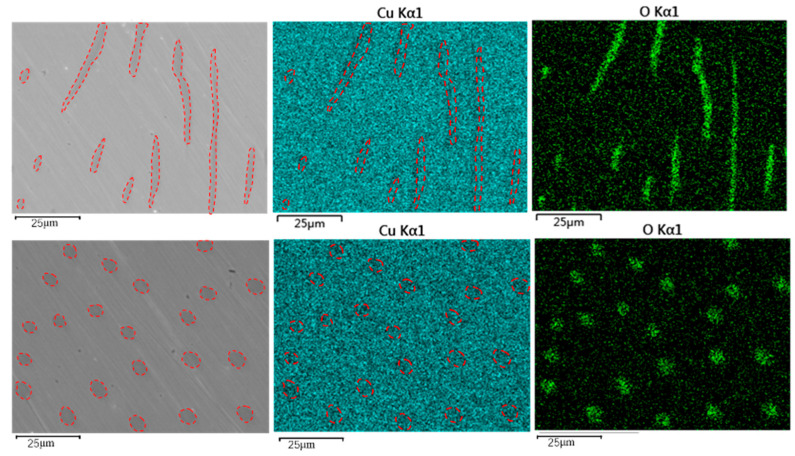
SEM morphology of microwave-sintered Cu-rGO composites.

Next, we observed the microstructures of the composite samples by SEM. As the magnification increased, we observed round, dot-shaped precipitates uniformly distributed on the sample surface (Figure 5). To determine the compositions of the precipitates, we characterized the surface of the sample by EDS elemental mapping (Figure 6). In addition to the matrix component Cu, we clearly observed oxygen (O) at the precipitates. Accordingly, the precipitates observed on the sample surface were deemed to be CuO.

### 3.4. Effect of Precipitates on Microhardness

**Figure 7:**
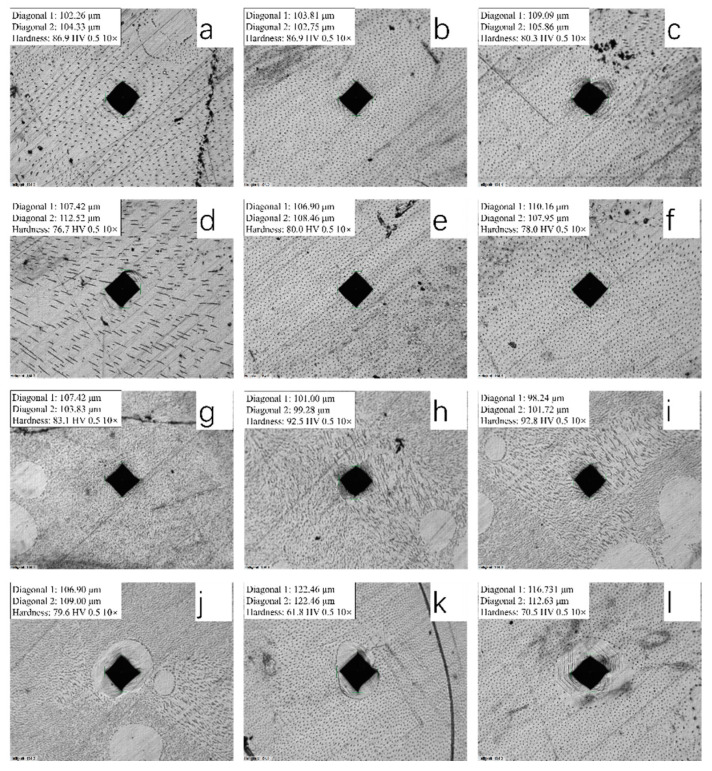
Sample microhardness (overall hardness values, **a**: 86.9 HV, **b**: 86.9 HV, **c**: 78 HV, **d**: 76.7 HV, **e**: 80 HV, **f**: 78 HV; eutectic areas, **g**: 83.1 HV, **h**: 92.5 HV, **i**: 92.8 HV; precipitation-free areas, **j**: 79.6 HV, **k**: 61.8 HV, **l**: 70.5 HV).

To further verify the properties of Cu-rGO composites, we used a microhardness tester to characterize different areas of the sample surface, and the results are presented in Figure 7a–f. The mean overall hardness of bulk composites prepared by microwave sintering of Cu-rGO composite powder was 81.1 HV. During the process of characterization, we found that different amounts of precipitates were present in different areas on the sample surface, and the microhardness values of these different areas varied somewhat. In addition, there were precipitate-free areas on the sample surface (Figure 7j–l), around which the CuO precipitates were distributed; these precipitate-free areas had a mean hardness of 70.6 HV. When measured at the points of elongated precipitates (Figure 7g–i), the mean hardness was 89.5 HV. The occurrence of this phenomenon was related to eutectic structures. The dot-shaped precipitates were hypoeutectic structures, whereas hypereutectic structures were generally rod-shaped; the microhardness of eutectic areas was 18.9 HV higher than that of precipitation-free areas, and 8.4 HV higher than the overall hardness. Therefore, we concluded that the eutectic structures were positively correlated with the microhardness of the composites, while precipitation-free areas were negatively correlated with the microhardness. Compared with pure Cu (71 HV), the overall hardness of the composites was increased 14.2% by microwave sintering with graphene oxide.

### 3.5. Electric and Thermal Conductivity

We then studied the effects of microwave sintering on the electrical and thermal conductivities of Cu-graphene composites. After microwave sintering, the electrical conductivity of the composites measured at five points was 50.59, 50.77, 50.61, 50.82, and 50.89 MS·m^−1^ (Table 1). The mean electrical conductivity was 50.74 MS·m^−1^, which corresponds to 87.10% IACS (i.e., 87.1% of the conductivity of annealed oxygen-free Cu). This high electrical conductivity implies that the Cu matrix and graphene could combine well.

The thermal conductivity of the composites was 391.62 W·m^−1^·K^−1^ (Table 1), while the thermal conductivity of pure copper is 397 W·m^−1^·K^−1^, indicating that the thermal conductivity of the composites was satisfactory. Because the lattice distortion in the Cu matrix induced by ball milling would hinder the thermal motion of electrons to a certain extent, graphene, which has excellent thermal conductivity, was used to compensate for this. Moreover, given the differing thermal expansion coefficients of graphene and the Cu matrix, and the association between the degree of thermal reduction and the dispersion of graphene, optimized processing conditions may increase the thermal conductivity of the composites.

## 4. Conclusions

In this study, Cu-rGO matrix composites were successfully prepared by microwave sintering of Cu-rGO composite powder. The composite powder, in which GO and Cu were evenly distributed and well combined, was obtained by wet ball milling.

The average microhardness of the microwave-sintered samples was 81.1 HV, which is 14.2% greater than that of pure Cu. During the preparation process, eutectic structures of CuO precipitated on the surface of the composites. The areas which contained precipitates had a higher microhardness of 89.5 HV, whereas the precipitate-free areas had a microhardness of 70.6 HV. Thus, the microhardness of the composites prepared by microwave sintering was positively correlated with eutectic structures.

The electrical conductivity of the microwave-sintered Cu-rGO matrix composite was 50.74 MS·m^−1^, corresponding to 87.10 % IACS, and the thermal conductivity was 391.62 W·m^−1^·K^−1^. The composites demonstrated favorable electrical and thermal conductivities, with electrical conductivity close to that of the international standard annealed oxygen-free Cu (58.25 MS·m^−1^ or 100% IACS) and thermal conductivity close to that of pure copper (397 W·m^−1^·K^−1^).

## Figures and Tables

**Table 1:** Electrical and thermal properties of Cu- rGO composites at room temperature.

Performance	Position					Mean Value		
Electrical conductivity	1	2	3	4	5	Electrical conductivity (MS·m^−1^)		IACS (%)
	5.059	50.77	50.61	50.82	50.89	50.74		87.1
Thermal properties	ρ/(g·cm^−3^) 8.56		a/(mm^2^·s^−1^) 91.36		Cp/(J·g^−1^·K^−1^) 0.49		λ/(W·m^−1^·K^−1^) 391.62	

## Data Availability

The data underlying this article will be shared on reasonable request from the corresponding author.
